# Jackfruit *(Artocarpus heterophyllus)* seed powder supplementation helps to maintain metabolic homeostasis in both normal and high-sugar diet-fed mice

**DOI:** 10.5455/javar.2025.l905

**Published:** 2025-06-16

**Authors:** Ohi Alam, Md. Kamrul Hasan Kazal, Romana Jahan Moon, Chayon Goswami, Rakhi Chacrabati

**Affiliations:** 1 Department of Biochemistry and Molecular Biology, Bangladesh Agricultural University, Mymensingh, Bangladesh; 2 Interdisciplinary Institute for Food Security, Bangladesh Agricultural University, Mymensingh, Bangladesh

**Keywords:** Diabetes, High sugar diet, Jackfruit seed powder, Obesity

## Abstract

**Objective:**

Diabetes mellitus and obesity stand as globally prevalent, life-threatening metabolic diseases. It has been reported that the intake of a high-sugar diet (HSD) increases the risk of developing diabetes and obesity. Therefore, it is necessary to find an alternative supplemental diet that could reduce the harmful consequences of excessive sugar consumption. The current study aimed to explore how jackfruit seed powder (JSP) could potentially prevent metabolic diseases in mice induced by an HSD.

**Materials and Methods:**

Swiss albino male mice were categorized into six groups fed normal and HSDs with or without JSP supplementation for 8 weeks. After the end of the feeding trial, different parameters related to glucose and lipid homeostasis were measured.

**Results:**

We found that 20% of JSP supplementation significantly decreased food intake and body weight gain induced by HSD. Supplementation of JSP reduced the liver weight, white adipose tissue, and brown adipose tissue weight in HSD-fed mice. Moreover, the addition of JSP with HSD improved the glucose tolerance test and reduced the levels of total cholesterol, triglyceride, and low-density lipoprotein cholesterol.

**Conclusion:**

These findings indicate that adding 20% JSP is particularly efficient in averting the onset of metabolic diseases induced by an HSD.

## Introduction

The rise in obesity is particularly associated with changes in eating and dietary patterns, for example, a preference for processed foods and beverages with added sugar and a sedentary lifestyle. Consumption of a high-sugar-enriched diet has increased dramatically all over the world and it is considered that excess consumption of a high-sugar diet (HSD) is a major contributing factor to the onset of obesity [1]. The prevalence of obesity has been rising globally during the past few decades [2]. Obesity is a risk factor for a broad range of diseases such as type 2 diabetes, insulin resistance, dyslipidemia, cardiovascular disease, and non-alcoholic fatty liver disease, which makes obesity one of the main public health concerns [3]. One of the main approaches for managing obesity is intensive lifestyle intervention that involves strict dietary restrictions and regular exercise. However, poor patient compliance often results in long-term weight loss [4,5]. Several anti-obesity drugs have been suggested for quick effect. Nevertheless, the current drugs have been associated with severe adverse effects, including stomachaches, insomnia, heart problems, vomiting, headaches, and constipation [6]. In recent years, natural plant-based remedies have gained attention as sustainable and safer alternatives for managing metabolic disorders.

Jackfruit (*Artocarpus heterophyllus lam*) is a tropical climacteric fruit belonging to the Moraceace family [7]. It holds the status of being Bangladesh’s national fruit and is known locally as “Kathal”. Jackfruit contains a high proportion of protein, easily digestible starch, essential minerals, and vitamins. It is regarded as an energy-rich fruit that is useful for treating physical or mental fatigue, stress, and weakness of muscle, as well as beneficial for athletes. It has been reported that jackfruit has diverse properties including antimicrobial, anti-diabetic, anti-inflammatory, antioxidant, and anthelminthic effects [8]. The seeds of a jackfruit constitute roughly 18%–25% of the fruit’s overall weight, with each fruit typically containing around 100–500 seeds [9]. Jackfruit seeds contain approximately 45.31%–58.38% moisture, 44.61%–54.69% dry matter, 3.63%–4.45% ash, 6.07%–7.32% protein, 0.39%–0.80% fat, 1.26%–1.71% fiber [10]. Jackfruit seeds also contain vitamin A, vitamin C, thiamin, and riboflavin [10]. Moreover, it is a good source of many important minerals, including N, P, K, Ca, Mg, S, Zn, Cu, and so on [11]. Jackfruit seed contains approximately 70%–85% total starch when dry, among which 25%–45% amylose and 45%–80% amylopectin. Due to the presence of a high amount of amylose, jackfruit starch is a low-digestible starch. Moreover, Jackfruit seed starches exhibit a greater proportion of resistant starch at approximately 75%, improved swelling ability, increased water absorption capacity, and a higher gelatinization temperature [12]. Essential fatty acids, such as linoleic and linolenic acids, which have a significantly greater antioxidant capacity, are also found in jackfruit seed [13]. Jackfruit seed reduces the risk of heart disease, promotes weight loss, and prevents constipation because of its high dietary fiber content [14].

Furthermore, jackfruit seeds’ resistant starch aids in blood sugar regulation and intestinal health maintenance. Resistant starch additionally aids in lowering blood cholesterol, reducing glycemic index, lessening bile stone formation, managing diabetes, and enhancing mineral absorption [15]. Seeds also possess two lectins (Artocarpin and Lectin) that provide immunological characteristics and serve as a means to assess the immune system of an individual with HIV infection [12]. In addition, jackfruit seeds possess several phytonutrients such as lignans, flavones, and saponins which have antioxidant, anticancer, antiulcer, antihypertensive, and antiaging effects [16]. Despite having promising health-beneficial findings of jackfruit seed, limited research has explored the specific effects of jackfruit seed powder (JSP) on metabolic disorders in experimental models. Furthermore, the comparative effects of JSP on normal versus HSD-fed mice remain unexplored. Therefore, to address this gap, the present study was carried out to investigate the effects of jackfruit seeds on metabolic parameters in both normal and HSD-fed mice.

## Materials and Methods

### Ethical approval

All procedures used in this research were authorized (AWEEC/BAU/2020_30) by the Animal Welfare and Experimentation Ethics Committee of Bangladesh Agricultural University, in accordance with the international standards outlined by the Council for International Organizations of Medical Sciences for animal-related biomedical research.

### Jackfruit seeds collection and preparation of powder

Ripen jackfruits were purchased from the nearby marketplace in Mymensingh, Bangladesh. After that, jackfruits were opened, and the seeds inside were taken out. These seeds underwent a thorough washing, slicing, and sun-drying process. Following the sun-drying, they were subjected to 24 h in a 60°C oven to eliminate excess moisture. After proper drying, the pieces were finely ground using a grinder and stored in a polythene bag for future use.

### Experimental animals

Six-week-old Swiss Albino male mice (initial body weight 30 ± 5 gm) were collected from the Animal Resources Facility at the International Centre for Diarrheal Disease Research, Bangladesh, and allowed to adapt to their new surroundings for a period of 12 days. The animals were kept in a well-ventilated environment maintained at a temperature of 28 ± 2°C, with a relative humidity level of 70%–80%, and subjected to natural day and night lighting conditions. Prior to the beginning of the feeding experiments, normal food and water were supplied *ad libitum*. The mice were separated into six groups comprising at least four individuals. To reduce potential stress reactions throughout the experiment, the animals were routinely handled throughout the acclimatization period.

### Experimental design

Two different doses (10% and 20%) of JSP were supplemented with both the normal diet (ND) and the HSD (supplemented with 30% sucrose). These are as follows:

Group A: ND-fed mice

Group B: ND-fed mice with 10% JSP (ND + 10% JSP)

Group C: ND-fed mice with 20% JSP (ND + 20% JSP)

Group D: HSD-fed mice

Group E: HSD-fed mice with 10% JSP (HSD + 10% JSP)

Group F: HSD-fed mice with 20% JSP (HSD + 20% JSP)

### Food formulation and diet paradigms

The ND was prepared by following the standard procedure previously described by Goswami et al. [17] with slight modification (Table 1). In this experiment, six diet paradigms were deployed: i) ND, ii) ND supplemented with 10% (w/w) of JSP (ND + 10% JSP), iii) ND supplemented with 20% (w/w) of JSP (ND + 20% JSP), iv) 30% (w/w) sucrose (HSD), v) HSD supplemented with 10% (w/w) of JSP (HSD + 10% JSP), and vi) HSD supplemented with 20% (w/w) of JSP (HSD + 20% JSP). The dosage justification was based on previous studies [18,19]. The diets were provided *ad libitum* for animals and changed daily to ensure their quality. Each treatment group consisted of a minimum of four mice housed individually in cages. These dietary interventions were consistently applied for 8 weeks as part of the treatment protocol.

**Table 1.** Ingredients of the experimental diets used in the study.

Food ingredients	ND	ND +10% JSP	ND +20% JSP	HSD	HSD + 10% JSP	HSD+ 20% JSP
Wheat	60%	50%	40%	30%	20%	10%
JSP	-	10%	20%	-	10%	20%
Sucrose	-	-	-	30%	30%	30%
Rice polished	5.5%	5.5%	5.5%	5.5%	5.5%	5.5%
Fish meal	10.0%	10.0%	10.0%	10.0%	10.0%	10.0%
Oil cake	6.0%	6.0%	6.0%	6.0%	6.0%	6.0%
Gram	0.39%	0.39%	0.39%	0.39%	0.39%	0.39%
Pulses	0.39%	0.39%	0.39%	0.39%	0.39%	0.39%
Milk	0.38%	0.38%	0.38%	0.38%	0.38%	0.38%
Soybean oil	1.5%	1.5%	1.5%	1.5%	1.5%	1.5%
Molasses	0.095%	0.095%	0.095%	0.095%	0.095%	0.095%
Salt	0.095%	0.095%	0.095%	0.095%	0.095%	0.095%
Embavit (vitamin)	0.1%	0.1%	0.1%	0.1%	0.1%	0.1%

### Measurement of food and water intake

The individual mouse food and water consumption were measured weekly at 10:00 a.m. for 8 weeks using the following formula:

Food intake = Weight of the supplied food − Weight of the remaining food

Water intake = Weight of the supplied water − Weight of the remaining water.

### Measurement of body weight

Each mouse’s weight was measured using an electric balance (eki300-2n electronic scale, A&D Company Ltd., Korea) every 7 days until the experiment was completed. The change in body weight (ΔBW) for each mouse was calculated at the end of the feeding experiment.

### Intraperitoneal glucose tolerance test (ipGTT)

At the end of the feeding trial, we conducted the ipGTT. Mice underwent a fasting period of about 8 h, placed in clean cages devoid of food or waste in the hopper or cage bottom, while maintaining access to drinking water. Using a fresh or sterilized scalpel blade, we scored the tip of the tail, discarding the initial small blood drop. Less than 5 μl of blood was applied onto the test strip of a standardized automated blood glucose meter (Glucoleader^TM^ Enhance Blood Glucose Meter, HMD Biomedical Inc., Hsinchu County, Taiwan). Each mouse received a single intraperitoneal injection of glucose (2 gm/kg BW). We recorded blood glucose levels at 0, 15, 30, 60, and 120-min post glucose injection for each mouse. Subsequently, we calculated the area under the curve (AUC) data based on the blood glucose levels observed in the ipGTT.

### Measurement of blood lipid profile

Lipid profile assessments included analyzing parameters like total cholesterol (TC) levels measured via the cholesterol oxidase-peroxidase-4-aminoantipyrine (CHOD-PAP) method [20], triglyceride (TG) levels assessed using the GPO-PAP method and high-density lipoprotein (HDL) cholesterol levels determined through the CHOD-PAP method [21]. The HumaTex febrile antigen test kit from Human Diagnostic in Wiesbaden, Germany was utilized, and the absorbance for all tests was measured using the Humalyzer, Model No-3000, from Human GmbH in Wiesbaden, Germany. Serum low-density lipoprotein (LDL) cholesterol concentrations were computed using the Friedewald equation [22] as outlined below:

LDL cholesterol (mg/dl) = TC – HDL cholesterol – (TG/5)

### Measurement of organs weight

Following blood collection, internal organs including the liver, heart, kidneys, white adipose tissue (WAT), and brown adipose tissue (BAT) were collected and trimmed to remove unnecessary tissue. These organs were rinsed in a saline solution and positioned on filter paper to eliminate surface moisture. Subsequently, the organ weights were measured using a digital balance (eki300-2n electronic scale, A&D company Ltd., Korea).

### Histopathological examination of liver

After sacrificing the animals, liver samples were collected immediately and fixed in 10% neutral buffer formalin for 72 h. These samples were subsequently processed to create paraffin sections and stained using hematoxylin and eosin, following the established protocol outlined by Sultana et al. [23]. In brief, the fixed tissues underwent a dehydration process using ethanol of varying concentrations and were then cleared with xylene before being embedded in paraffin. Furthermore, the specimens were sliced using a rotary microtome and subjected to hematoxylin and eosin staining. The histological slides of the liver were examined under a microscope, and distinct features from each slide were captured using an integrated microscope camera. In each experimental group, a total of five microscopic fields were assessed from each tissue section.

### Statistical analysis

Prism 5 (GraphPad Software 7.0, CA) was used for all statistical analyses. Data were presented as mean ± SE. To identify significant differences among treatment groups, an analysis of variance (ANOVA) was performed, followed by Tukey’s *post hoc* test. A significance threshold of *p* < 0.05 was set for all analyses.

## Results

### Food intake

**Figure 1.**
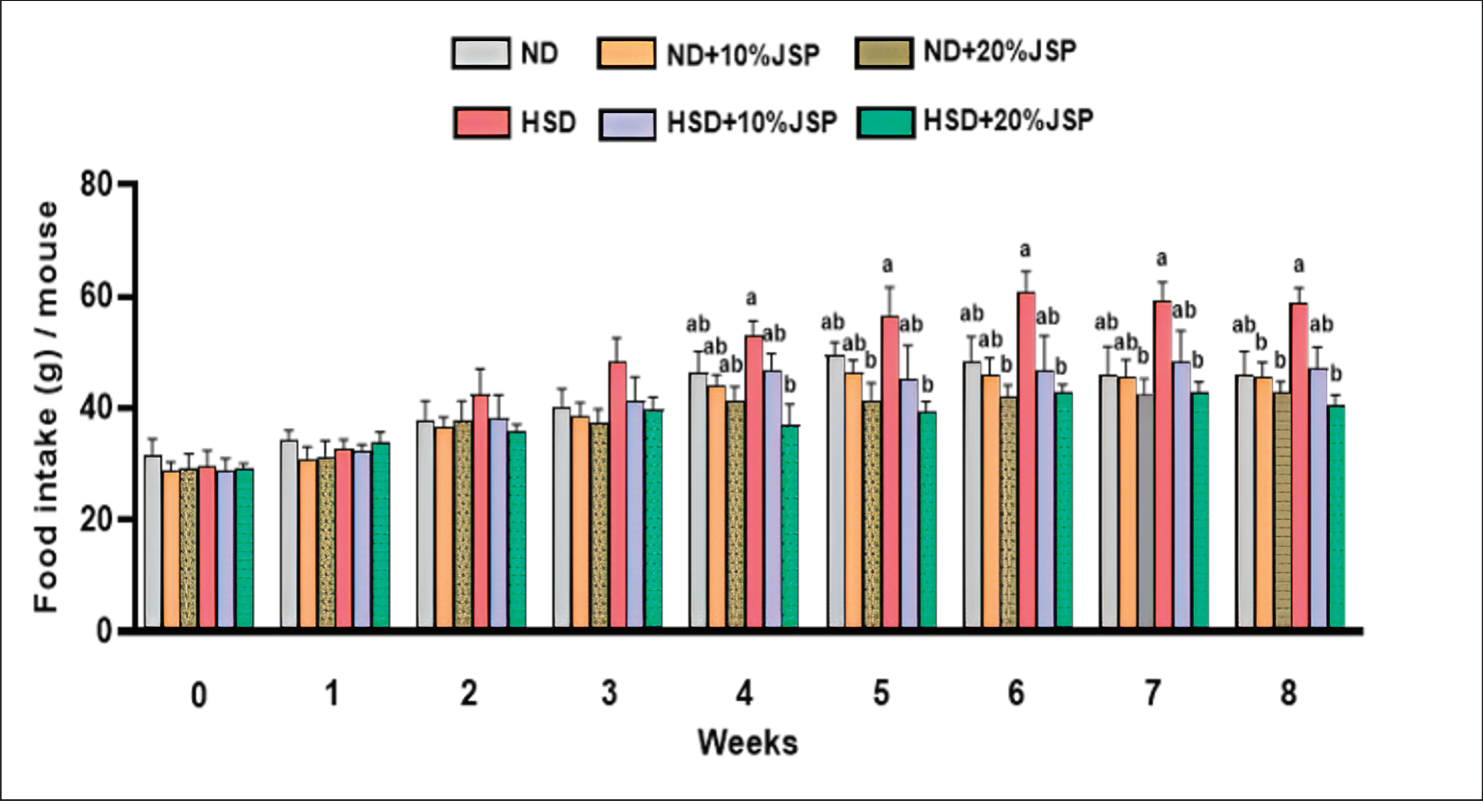
Supplementation of JSP decreased food intake in HSD diet-fed mice. Data were expressed as mean ± SEM (*n* ≥ 3 mice/group) and statistically different at *p* < 0.05 by one-way ANOVA followed by Tukey’s *post hoc* test. Bars having different letters indicate statistical differences among the groups. HSD = high-sugar diet; JSP = jackfruit seed powder; ND = normal diet.

Food intake was measured on a weekly basis throughout the entire feeding experiment. Initially, there were no differences in food intake among the groups. However, the HSD group showed a growing trend in food intake from the 2nd to the 8th week. The addition of JSP at a dose of 10% and 20% with HSD showed a decrease in food intake (Fig. 1). Food consumption in the ND-fed group was slightly higher than that of the JSP-supplemented groups over the whole experimental period, and the addition of JSP to the ND curbed food consumption. By the 4th week, food consumption of the HSD group was notably higher than the JSP-supplemented groups, and these JSP-supplemented groups reduced food intake in a dose-dependent manner. Specifically, the HSD + 20% JSP group exhibited a significant reduction in food intake, whereas the HSD + 10% JSP group failed to show the same reduction. From the 5th week to the 8th, a similar trend of food intake was noticeable, where the HSD group consumed more food than the other groups. However, JSP supplementation reduced food intake, and a significant reduction was observed in the HSD + 20% JSP group. Though the HSD + 10% JSP group showed a remarkable decrease, it statistically insignificant when compared to the HSD group.

### Water intake

**Figure 2.**
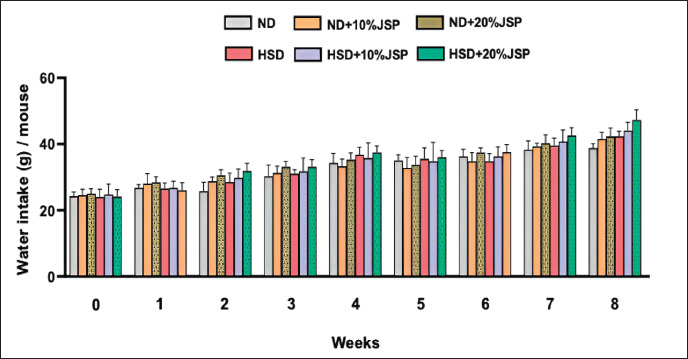
JSP supplementation increased the water intake of mice. Data were expressed as mean ± SEM (*n* ≥ 3 mice/ group) and statistically different at *p* < 0.05 by one-way ANOVA followed by Tukey’s *post hoc* test. HSD = high-sugar diet; JSP = jackfruit seed powder; ND = normal diet.

The findings revealed that there was no discernible difference in water consumption during the initial phase of the experiment (Fig. 2). As the experiment progressed, it became evident that supplementation of JSP into both a HSD and a ND led to a gradual increase in water intake from the 2nd to the 8th week, with some fluctuations. From the 2nd and 3rd weeks, a similar pattern of water consumption was observed where the JSP-supplemented groups consumed more water than the ND and HSD groups, but the values were statistically insignificant. During the 4th week of the experiment, the ND + 10% JSP group consumed slightly less water than did the control group. Similarly, the HSD + 10% JSP group also consumed less water than the HSD group. In 5th week, the water intake of the control group (ND) was slightly higher than that of the JSP-supplemented groups, whereas the water intake of the HSD + 10% JSP group was lower than that of the HSD group; however, the water intake of the HSD + 20% JSP group was higher than that of the HSD group. During the last 2 weeks of the study, the JSP-supplemented groups consistently exhibited higher water intake compared to both the ND and HSD groups. This indicates that JSP supplementation had an influence on water consumption.

### Body weight

**Figure 3.**
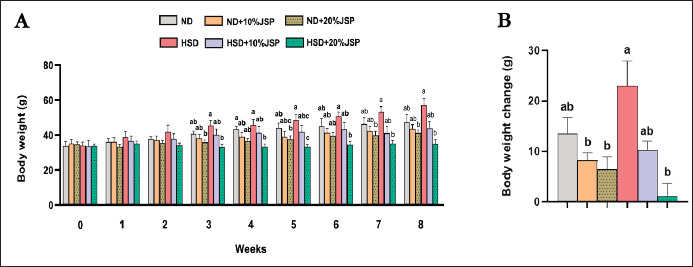
JSP addition with a high sugar diet reduced the body weight gain of mice. (A) Body weight. (B) Body weight gain was measured at the end of the experiment. Data were expressed as mean ± SEM (*n* ≥ 3 mice/group) and statistically different at *p* < 0.05 by one-way ANOVA followed by Tukey’s *post hoc* test. Results having different letters indicate statistical differences among the groups. HSD = high-sugar diet; JSP = jackfruit seed powder; ND = normal diet.

Present findings revealed that the body weight of the JSP-supplemented groups was lower than the HSD group, but it was not statistically significant until 2nd week. However, starting from the 3rd week until the 8th week, JSP supplementation consistently reduced the body weight of mice in a dose-dependent manner. Specifically, the HSD + 20% JSP group showed a significant decrease, while the HSD + 10% JSP group exhibited a decrease that was not statistically significant when compared to the HSD group (Fig. 3A). Similarly, both the ND + 10% JSP and ND + 20% JSP groups also experienced a continual decrease in body weight compared to the ND group from the 3rd week until the end of the trial, although these changes were not statistically significant. Notably, the body weight gain in the HSD group was higher than in the other experimental groups. However, the addition of a 20% JSP supplement to the HSD diet significantly mitigated the body weight gain caused by HSD. Additionally, the body weight gains in the 10% and 20% JSP-supplemented groups and the ND group were comparable.

### Glucose tolerance test

**Figure 4.**
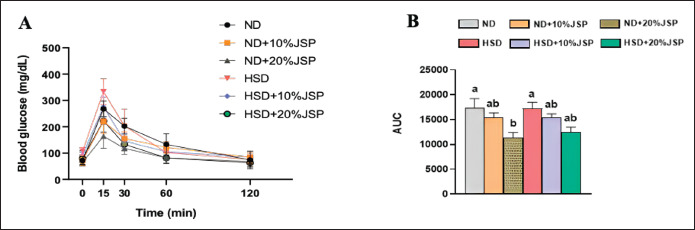
JSP supplementation improved glucose tolerance test in HSD–fed mice. (A) Blood glucose levels after an intraperitoneal glucose injection (2gm/kg BW). (B) Area under curve (AUC) in GTT test. Data were expressed as mean ± SEM (*n* ≥ 3 mice/group) and statistically different at *p* < 0.05 by one-way ANOVA followed by Tukey’s *post hoc* test. Bars having different letters indicate statistical differences among the groups. HSD = high-sugar diet; JSP = jackfruit seed powder; ND = normal diet.

At the end of the feeding trial, the glucose tolerance test (GTT) was conducted to evaluate the blood glucose homeostasis. The results from GTT demonstrated that the HSD group had a high level of blood glucose as compared with the ND group, particularly after 15- and 30-min glucose injections (2 gm/kg BW). Supplementation of JSP into HSD, particularly 20% JSP but not 10% JSP, showed a considerable decrease in blood glucose levels as compared to the HSD group (Fig. 4A). The blood glucose levels of ND + 10% JSP and ND + 20% JSP were lower than that of the ND group at all-time points. Moreover, the AUC derived from GTT data revealed that the AUC of the JSP-supplemented groups (10% and 20%) was low in comparison to the HSD group, though the values were not statistically significant (Fig. 4B). The AUC of the (ND + 20% JSP) group was significantly lower when compared to the ND group, whereas the AUC of the (ND + 10% JSP) group was comparable with the ND group.

### Organ weight

**Figure 5.**
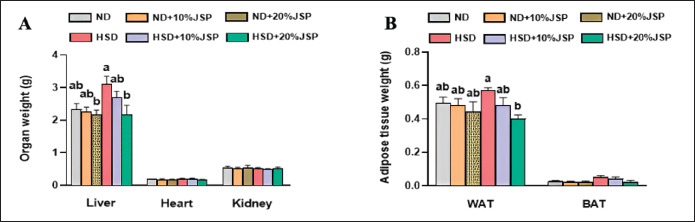
JSP supplementation decreased the liver weight, WAT and BAT weight in HSD-fed mice. (A) Weight of liver, heart, and kidney, (B) weight of brown adipose tissue (BAT), white adipose tissue (WAT). Data were presented as mean ± SEM (n ≥ 3 mice/group) and statistically different at *p* < 0.05 by one-way ANOVA followed by Tukey’s *post hoc* test. Results having different letters indicate statistical differences among the groups. HSD = high-sugar diet; JSP = jackfruit seed powder; ND = normal diet.

The liver, kidney, heart, WAT, and BAT were collected and measured following the collection of the blood sample. The results demonstrated that the HSD diet increased the liver weight, and supplementation of JSP with the HSD diet at a dose of 20% significantly decreased the liver weight (Fig. 5A). The HSD + 10% JSP also showed a remarkable decrease in liver weight, but this was not statistically significant when compared to the HSD group. Moreover, the liver weight of the JSP-supplemented groups was lower than that of the control group, and a prominent decrease was noticeable in the ND + 20% JSP group, although the difference was not statistically significant. The heart and kidney weights were almost similar among the groups. HSD-fed mice had higher adipose tissue weight, which was significantly reduced by 20% JSP supplementation (Fig. 5B). Although 10% JSP reduced the weight of WAT, it was not significant compared to the HSD group. The weight of BAT was similar among the groups; however, the HSD group tended to have a higher BAT weight, and the incorporation of JSP reduced the BAT weight.

### Liver histological changes

**Figure 6.**
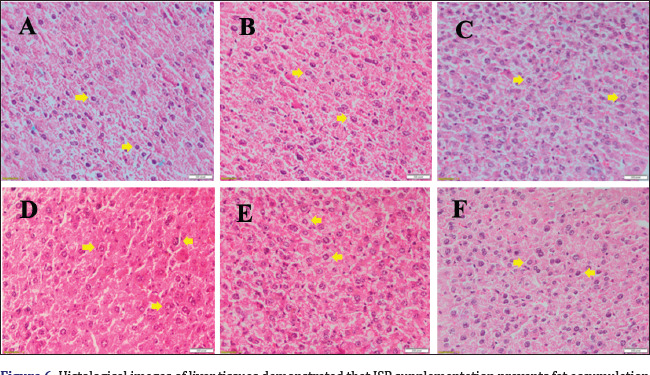
Histological images of liver tissues demonstrated that JSP supplementation prevents fat accumulation in HSD-induced obese mice. No histological abnormalities were observed in normal diet-fed mice with or without JSP supplementation (A–C). Mice fed with HSD for 8 weeks develop the initial stage of hepatic steatosis, showing irregular cell arrangement, enlarged cell size, and nuclei (D). Supplementation of JSP at a dose of 10% and 20% resulted in the prevention of fat deposition in hepatocytes, showing regular size and arrangement of cell and nuclei (E, F). (A) ND group, (B) ND + 10%JSP group, (C) ND + 20% JSP group, (D) HSD group, (E) HSD + 10% JSP group, and (F) HSD + 20% JSP group. The liver tissues were subjected to histological study by staining with hematoxylin and eosin (H&E). All magnifications: ×40. The yellow arrows indicate cell nuclei.

Hematoxylin and eosin staining of liver tissue sections (observed at a 40× magnification) showed well-organized cells with distinct and easily visible nuclei in the ND group (Fig. 6A). However, the liver morphology of the HSD group showed an irregular cell arrangement characterized by enlarged cell size and nuclei (Fig. 6D), suggesting an initial phase of fat buildup, despite the absence of cytoplasmic vacuoles. Comparatively, smaller cell size and nuclei were observed in the (HSD + 20% JSP) group (Fig. 6E) which indicates JSP prevents fat accumulation in liver tissues.

### Blood lipid profile

**Figure 7.**
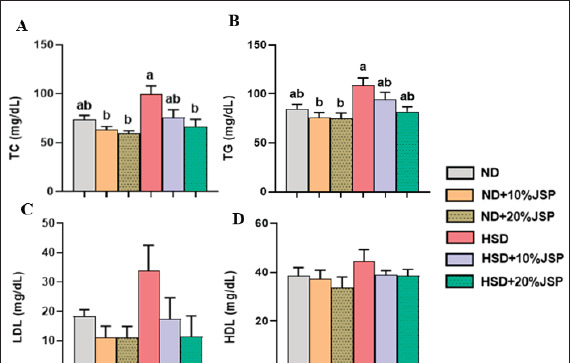
JSP supplementation improved blood lipid profiles. (A) Total cholesterol (TC), (B) Triacylglycerol (TG), (C) Low-density lipoprotein (LDL), and (D) High-density lipoprotein (HDL). Data were expressed as mean ± SEM (n ≥ 3 mice/group) and statistically different at *p* < 0.05 by one-way ANOVA followed by Tukey’s *post hoc* test. Bars having different letters indicate statistical differences among the groups. HSD = high-sugar diet; JSP = jackfruit seed powder; ND = normal diet.

At the end of the experiment, serum samples were collected and lipid profiles were assessed. The findings showed that mice fed HSD tended to have higher levels of TC, TG, LDL, and HDL. In comparison to the HSD control group, the 20% JSP-supplemented group demonstrated a significant decrease in TC content, whereas the HSD + 10% JSP group exhibited an insignificant decrease (Fig. 7A). Additionally, the TC levels in the JSP-supplemented groups were marginally lower than those in the control group (ND). TG concentrations were higher in the HSD group than in the ND group, and the inclusion of JSP with HSD reduced TG levels (Fig. 7B). Although the 20% JSP group showed a considerable reduction in TG level, the value was not significant when compared to the HSD group. Serum LDL level was remarkably high in the HSD group in comparison to the other groups. However, the incorporation of JSP into HSD decreased LDL concentration, although the difference was not statistically significant (Fig. 7C). There were no significant variations observed in serum HDL levels among the group (Fig. 7D).

## Discussion

The results of the study demonstrated that JSP was beneficial in lowering food intake, elevated body weight gain, and the dysregulation of blood glucose homeostasis induced by HSD. The addition of JSP with HSD also curbed the weight of the liver, and WAT. Furthermore, supplementation of JSP showed a remarkable decrease in the concentration of serum TC, triacylglycerol, and LDL. In the present study, we observed that HSD-treated mice consumed more food than the ND fed mice. Supplementation of JSP at a dose of 10% and 20% in HSD curbed the food intake. Notably, 20% of JSP supplementation exhibited a significant decrease in food intake. Reduction in food consumption is associated with the complex interaction among neural, endocrine, gastrointestinal, and adipose tissues that regulate satiety and hunger [24]. Moreover, the properties of dietary fiber may impact postprandial satiety in animals via modulating digestive physiology [25].

Dietary fiber can produce viscous and/or jelly-like substances in the colon, slowing stomach emptying and simultaneously increasing stomach distension, which may activate afferent vagal signals of fullness [26]. In the current study, JSP supplementation reduced food intake of mice may be due to the presence of its dietary fiber which reduces gastric emptying rate that eventually prolongs the satiety state and decreases food intake. Reduction in food intake means reduce energy intake [27]. The potential way in which a substance might counteract the onset of diabetes and obesity could primarily involve a decrease in food consumption. Therefore, it is improbable that the alleviation of diabetes and obesity symptoms through the use of JSP can be attributed to a reduction in food intake.

Our study also demonstrated that JSP-supplemented groups consumed more water compared to the ND and HSD groups. Furthermore, a slightly greater intake of water was observed in the HSD + 20% JSP group. Increased water consumption is often associated with a feeling of thirst, which typically occurs when a diet contains a high content of dietary fibers with a strong water-binding capacity [28]. Therefore, we can infer that the higher water intake in JSP-supplemented groups may be attributed to the mice consuming a greater amount of fiber. Consumption of sugar is associated with reduced diet quality, increased energy intake [29], and also increased the prevalence of obesity. A Previous study reported that increased consumption of sweetened beverages such as soft drinks, commercially available fruit juice, and fast foods is thought to be a factor in childhood and adolescent weight gain as well as the risk of obesity [30]. In another study, researchers reported that an increased intake of sweetened beverages is connected to weight gain and an increased risk of metabolic diseases such as diabetes [31].

Our current findings also revealed that mice fed a HSD exhibited greater weight gain compared to the other groups after an 8-week treatment period. This weight gain, however, was decreased by 10% and 20% JSP supplementation in their diet. Notably, significant body weight gain reduction was observed in the 20% JSP-supplemented group. The potential mechanism by which JSP reduces body weight gain may be attributed to its dietary fiber content. Dietary fiber can help either maintain or reduce body weight by promoting a feeling of fullness and reducing calorie intake [32] decreasing the absorption of macronutrients [33], slowing down the digestion of starch, and stimulating the release of gut hormones [18]. Furthermore, epidemiological studies have revealed that dietary fiber is linked to lower body weight and a decreased risk of weight gain [34].

In the present study, we also observed that mice fed an HSD had a greater liver weight compared to the control group (ND). The addition of 20% JSP with HSD demonstrated a significant reduction in liver weight. In contrast, adding 10% JSP resulted in a minor and statistically insignificant reduction in liver weight. These findings suggest that JSP effectively counteracts liver enlargement. JSP supplementation also reduced the weight of WAT and BAT. The exact mechanism by which JSP decreased the weight of adipose tissue is unknown. A possible mechanism could be that consumption of JSP influences the rate of hepatocyte apoptosis as it is a good source of dietary fiber. A previous study demonstrated that rats fed dietary fiber had lower rates of hepatocyte apoptosis, which in turn led to lower levels of abdominal fat formation [35]. Moreover, polyphenols such as saponins present in JSP may have an effect on pancreatic lipase activity. Previous studies demonstrated that saponins effectively inhibit pancreatic lipase activity both *in vitro* and *in vivo*, suggesting a role in hindering the intestinal absorption of dietary fat [36].

These findings highlight the effectiveness of JSP in preventing fat accumulation and ultimately mitigating mice’s body weight gain. One of the drawbacks of our study is that we did not identify the specific chemical component in jackfruit seeds responsible for these observed physiological effects. Glucose tolerance indicates the ability of an individual to withstand an oral glucose challenge, which necessitates a rise in insulin secretion from islet β cells and insulin-mediated augmentation of peripheral tissue glucose uptake [37]. Glucose intolerance develops when a person cannot respond to the glucose load from a meal. It has been reported that excessive consumption of HSDs can damage pancreatic islet β cells and lower insulin sensitivity, which can lead to glucose intolerance [38]. In this study, we found that HSD-fed mice demonstrated an impaired resistance to glucose (2 gm/kg BW) challenge. However, the addition of JSP with HSD noticeably improved glucose tolerance in mice. The improvement in GTT by JSP supplementation with HSD could be related to the high dietary fiber and resistant starch content of JSP. A previous study reported that dietary fibers such as resistant starch, β-glucan, inulin, and so on, had favorable effects on the regulation of postprandial plasma glucose level [39].

Hyperlipidemia is a significant contributor to global mortality [40]. The intake of added sugars is linked to adverse effects on lipid profiles, including elevated TG levels, TG-to-HDL cholesterol ratios, and TC-to-HDL cholesterol ratios, thereby increasing the susceptibility to dyslipidemia [41,42]. Excess consumption of sugar increased the TG concentration by triggering hepatic *de novo* lipogenesis [43]. In the present study, as expected, HSD-fed mice showed an increasing trend in the concentration of TC, TGs, and LDL in the blood. However, supplementation of JSP with HSD cured the TC, TG, and LDL levels in a dose-dependent manner. Supplementation of 20% JSP exhibited a significant decrease whereas 10% JSP supplementation showed an insignificant reduction in the level of TC. The concentration of TG was insignificantly lower in the JSP-treated mice than that of the HSD group. Moreover, 20% JSP supplementation considerably reduced the LDL level in comparison to the HSD group, though the values were not statistically significant. The concentration of HDL was slightly lower in JSP-supplemented groups than in the HSD-fed mice. Lipid-lowering properties of JSP may be attributed to a higher amount of dietary fiber which lowers cholesterol due to its high viscosity, water holding capacity and adsorption capacity.

Luo et al. [44] reported that dietary fiber reported that dietary has the ability to bind or bile acids and cholesterol, resulting in a reduction in cholesterol. Moreover, JSP contains flavonoids and phenolic compounds that may enhance lipid metabolism. Although the beneficial effects of JSP were observed in the present study, the active ingredients that specifically regulate the metabolic processes and their mechanism of action are not identified. Further research is necessary to identify these specific constituents, which would enhance the scientific basis for using JSP as a therapeutic agent against metabolic disorders.

## Conclusion

The incorporation of JSP, especially at a 20% concentration, effectively alleviated excessive food intake and weight gain induced by HSD consumption. Additionally, supplementation with JSP led to decreased weights of the liver, WAT, and BAT. Furthermore, the inclusion of JSP in HSD decreased the levels of TC, TGs, and LDL cholesterol, while also assisting in maintaining normal blood sugar levels in mice fed HSD. Therefore, jackfruit seeds could serve as a promising dietary supplement to combat metabolic disorders caused by HSD.

## List of abbreviations

ANOVA, analysis of variance

AUC, area under curve

BW, body weight

BAT, brown adipose tissue

GTT, glucose tolerance test

HDL, high-density lipoprotein

HSD, high sugar diet

ipGTT, intraperitoneal glucose tolerance test

JSP, Jackfruit seed powder

LDL, low-density lipoprotein

ND, normal diet

TC, total cholesterol

TG, triglyceride

WAT, white adipose tissue.
